# Effectiveness and Safety of Sacituzumab Govitecan in Real-World Clinical Practice in Patients with Metastatic Triple-Negative and HR+/HER2-Negative Breast Cancer

**DOI:** 10.3390/biomedicines13092059

**Published:** 2025-08-23

**Authors:** Fernando Lago-Ballester, Adrián Martínez-Orea, Ana Laorden-Carrasco, María Sacramento Díaz-Carrasco, José Carlos Titos-Arcos, María Carmen Mira-Sirvent, Ginés Luengo-Gil, Mónica Martínez-Penella

**Affiliations:** 1 Hospital Pharmacy Department, Hospital General Universitario Santa Lucía, 30202 Cartagena, Spain; 2 Health Sciences Faculty, Universidad Católica de Murcia (UCAM), 30107 Guadalupe, Spain; 3 Hospital Pharmacy Department, Hospital General Universitario Morales Meseguer, 30008 Murcia, Spain; 4 Hospital Pharmacy Department, Instituto Murciano de Investigación Biosanitaria Pascual Parrilla (IMIB), Hospital Clínico Universitario Virgen de La Arrixaca, 30120 Murcia, Spain; 5 Group of Molecular Pathology and Pharmacogenetics, Hospital Pharmacy and Pathology Department, Instituto Murciano de Investigación Biosanitaria (IMIB), Hospital General Universitario Santa Lucía, 30202 Cartagena, Spain

**Keywords:** sacituzumab govitecan, triple-negative breast cancer, metastatic breast cancer, HER2-negative, real-world evidence, progression-free survival, overall survival

## Abstract

**Background/Objectives:** Sacituzumab govitecan (SG) is an antibody–drug conjugate targeting Trop-2 that has demonstrated clinical benefits in randomised trials for patients with metastatic triple-negative breast cancer (mTNBC) and metastatic hormone receptor-positive/HER2-negative (HR+/HER2− mBC) disease. However, real-world data on its effectiveness and safety are limited, especially in patients with poor performance status or central nervous system (CNS) involvement. This study aimed to evaluate the real-world outcomes of SG in these two subtypes. **Methods:** We conducted a retrospective, multicentre, observational study across three tertiary hospitals in Spain. Patients with mTNBC or HR+/HER2− mBC treated with SG between June 2022 and March 2025 were included. Clinical data, treatment history, adverse events (AEs), and survival outcomes were also recorded. The median progression-free survival (mPFS) and median overall survival (mOS) were estimated using Kaplan–Meier analysis. Univariate and multivariate analyses were performed to identify the factors influencing outcomes. The association between granulocyte colony-stimulating factor (G-CSF) prophylaxis and neutropenia was assessed using Fisher’s exact test. **Results:** A total of 56 patients were included in this study (33 with mTNBC and 23 with HR+/HER2− mBC). In the mTNBC group, mPFS was 4.0 months (95% CI: 1.94–5.98) and mOS was 11.0 months (95% CI: 4.80–17.12). In the HR+/HER2− mBC group, mPFS was 3.7 months (95% CI: 2.02–5.44) and mOS was 20.2 months (95% CI: 3.9–36.5). Fatigue, neutropenia, and gastrointestinal toxicity were the most common AEs. Primary G-CSF prophylaxis was not associated with a reduced incidence of neutropenia (*p* = 0.434). **Conclusions:** In routine practice, SG shows effectiveness comparable to that of randomised trials across both subtypes, with a safety profile consistent with pivotal studies. The observed toxicity profile was consistent with that described in pivotal clinical trials and other studies. The prophylactic use of G-CSF was not associated with an impact on the occurrence of neutropenia, but the incidence of neutropenia was lower than that in clinical trials and other studies that did not administer G-CSF prophylactically.

## 1. Introduction

Breast cancer is the most common neoplasm in women worldwide, with one-quarter of cases diagnosed before the age of 50 years in Western countries, and less than 5% before the age of 35 [1]. It is classified into multiple subtypes defined by immunohistochemical (IHC) testing of progesterone receptors (PR), oestrogen receptors (ER), human epidermal growth factor receptor 2 (HER2), and Ki67. This molecular classification, which divides breast cancer into three subtypes—mTNBC, HR+/HER2−, and HR+/HER2+—allows for the identification of subtypes with distinct therapeutic and prognostic profiles [2].

mTNBC accounts for approximately 15–20% of all breast cancers and is characterised by aggressive behaviour with high invasiveness, leading to elevated relapse rates, high mortality, poor prognosis, and limited targeted treatment options owing to the lack of PR, ER, and HER2 expression [3,4,5]. Because of the lack of molecular targets, chemotherapy remains the first-line standard of care, although its effectiveness is limited, particularly in the metastatic setting. This has driven the development of novel therapeutic approaches, such as immune checkpoint inhibitors such as atezolizumab and pembrolizumab in combination with chemotherapy, or antibody–drug conjugates (ADCs) such as SG, which target trophoblast cell surface antigen 2 (Trop-2) [3,4,5].

The HR+/HER2− subtype accounts for approximately 70% of breast cancer cases, making it the most prevalent subtype [6,7]. It is characterised by positivity for ER and PR and negativity for HER2, and is confirmed as negative by in situ hybridisation (ISH) techniques [7]. A distinct subtype of HR+/HER2− breast cancer, has recently been defined and characterised by HER2 IHC 1+ or 2+ and ISH− status, referred to as HER2-low breast cancer. The treatment of HR+/HER2− metastatic breast cancer (mBC) includes endocrine therapy (ET) with ER modulators or aromatase inhibitors (AIs) in combination with cyclin-dependent kinase (CDK) 4/6 inhibitors and PI3K/AKT/mTOR pathway inhibitors alongside ET. For metastatic disease refractory to endocrine therapy and CDK 4/6 and PI3K/AKT/mTOR inhibitors, treatment has traditionally been limited to chemotherapy, thereby reducing the therapeutic options. This has translated into an overall survival (OS) of less than 3 years and a 5-year survival rate of only 27%. In this context, SG has emerged as the only antibody–drug conjugate (ADC) approved for the treatment of HR+/HER2− mBC, allowing cytotoxic agents to be delivered directly to tumour cells via specific antibodies, and has demonstrated a significant benefit in progression-free survival (PFS) and response duration in patients with HR+/HER2− mBC [6,7].

SG is an antibody–drug conjugate (ADC) that combines an antibody targeting Trop-2, highly expressed in both aforementioned tumour subtypes, with the active metabolite of irinotecan (SN-38), linked via a hydrolysable linker [8,9]. SG is currently approved in Europe for the treatment of mTNBC and HR+/HER2− metastatic breast cancer (mBC) in patients who have received certain prior treatments, with a dosing schedule of 10 mg/kg administered on days 1 and 8 of each 21-day cycle as specified in the European Public Assessment Report of SG (EMA Trodelvy European Public Assessment Report link, https://www.ema.europa.eu/en/documents/product-information/trodelvy-epar-product-information_en.pdf (accessed on 11 August 2025)). Trop-2 is considered a key factor in TNBC, promoting epithelial–mesenchymal transition, facilitating metastatic spread, and conferring resistance to apoptosis. Overactivation is associated with highly aggressive phenotypes, including inflammatory involvement of lymph nodes, metastatic invasion, and reduced overall survival (OS) [10]. Trop-2 overexpression has been observed in 95% of primary TNBC tumours and 88% of their metastatic lesions [11], positioning Trop-2 as a relevant therapeutic target. Trop-2 expression has been studied in different breast cancer subtypes. In a study assessing Trop-2 expression in 94 breast cancers (luminal A, luminal B, HER2-positive, and triple-negative subtypes), no statistically significant differences in Trop-2 expression were observed between the groups. Intermediate-to-high Trop-2 levels have been identified in most tumours within each subtype, with wide intragroup variability and only exceptional cases showing low or absent expression [12]. The efficacy of SG in mTNBC and HR+/HER2− mBC appears to be consistent, regardless of high or low Trop-2 expression in these cancer types, as demonstrated in the ASCENT and TROPiCS-02 studies, where no statistically significant differences in PFS or OS were observed between the high- and low-expression groups [9].

In the phase III ASCENT clinical trial, SG demonstrated a significant improvement in median progression-free survival (mPFS), with 4.8 months for SG versus 1.7 months for the chemotherapy group, and a median overall survival (mOS) of 11.8 months for SG compared with 6.9 months for chemotherapy in previously treated patients with mTNBC [8]. Similarly, in the phase III TROPiCS-02 study, SG showed a clinically meaningful benefit over chemotherapy in patients with HR+/HER2− mBC who were refractory to multiple prior lines of therapy including CDK4/6 inhibitors. The mOS was 14.4 months in the SG group [95% CI 13.0–15.7] compared with 11.2 months in the chemotherapy group [10.1–12.7], and the mPFS was 5.5 months for SG versus 4.0 months for chemotherapy [9].

Despite the favourable mPFS and mOS outcomes of SG compared to chemotherapy in the ASCENT and TROPiCS-02 clinical trials for the treatment of mTNBC and HR+/HER2− mBC, evidence generated under controlled conditions is not always transferable to routine clinical practice. In real-world settings, the patient population is more heterogeneous and often presents with poorer performance status on the Eastern Cooperative Oncology Group (ECOG) scale. Patients with ECOG ≥ 2 were excluded from both the ASCENT and TROPiCS-02 trials, as well as those with multiple comorbidities and metastatic involvement that were also excluded from clinical trials, such as central nervous system (CNS) metastases. Therefore, real-world data are needed to validate the effectiveness and safety of SG in more complex and diverse clinical scenarios. Real-world studies evaluating the effectiveness and safety of SG remain limited, as is the number of patients included in these analyses. Notable examples include the multicentre studies by Alaklabi et al. and Hanna et al., both focused on patients with mTNBC [13,14]. In contrast, there is a clear lack of real-world data on the use of SG in patients with HR+/HER2− mBC, with only the clinical trials TROPiCS-02 and EVER-132-002, and the real-world study by Muğlu et al. available to date [9,15,16].

Despite these advances, real-world evidence on the efficacy and safety of SG in mTNBC, particularly HR+/HER2− mBC, remains limited, with a lack of data in patients with high ECOG scores or CNS involvement.

This study aimed to evaluate the effectiveness, including progression-free survival (PFS) and overall survival (OS), as well as the safety profile of SG in real-world settings by assessing the incidence of adverse events (AEs) and determining the potential impact of administering granulocyte colony-stimulating factor (G-CSF) as primary prophylaxis on the development of neutropenia secondary to SG treatment in clinically complex patients with mTNBC and HR+/HER2− mBC.

## 2. Materials and Methods

To address our research objectives, we conducted an observational, retrospective, multicentre study involving 56 patients carried out in three tertiary hospitals in Spain, as detailed below.

Patients diagnosed with mTNBC and HR+/HER2− mBC were included in this study. All patients who received at least two cycles of SG (Trodelvy; Gilead Sciences Ireland UC, Carrigtohill, County Cork, Ireland) were considered eligible. Those without available clinical follow-up data or who had received only a single SG administration were excluded.

Clinical and treatment data were collected for eligible patients who received SG between 24 June 2022 and 28 March 2025 across the three participating hospitals.

The sociodemographic variables included age at the start of SG treatment; ECOG performance status; location of metastases; prior treatments; primary prophylactic use of granulocyte colony-stimulating factor (G-CSF) on days 2, 3, 4, 9, 10, and 11 of each cycle at a fixed dose of 300 mg administered subcutaneously; recorded adverse events (AEs); and survival data such as treatment start and end dates, death, progression-free survival (defined as the time from the start of SG to radiological or clinical progression), and overall survival (defined as the time from the start of SG until death).

All statistical analyses were performed using SPSS software v29.0.2 (IBM Corp., Armonk, NY, USA). A descriptive statistical analysis was conducted, and the median progression-free survival (mPFS) and median overall survival (mOS) were estimated using the Kaplan–Meier method and log-rank test. Fisher’s exact test was used to compare the incidence of neutropenia between the patients who received G-CSF prophylaxis and those who did not during SG treatment. A univariate analysis of OS was performed to assess the influence of various variables on overall survival. The covariates included in the analysis were molecular subtype, ECOG performance status, location of metastases, prior treatment regimens, and G-CSF administration. Variables that showed a statistically significant association in the univariate analysis (*p* < 0.1) were included in a multivariate Cox regression model to identify the independent predictors of survival. Hazard ratios (HRs) with corresponding 95% confidence intervals (95% CI) were estimated for each variable included in the multivariate model. No prespecified comparative subgroup analyses of survival were performed. Additionally, exploratory descriptive subgroup analyses of neutropenia were performed, stratified by age (<60 vs. ≥60 years), ECOG performance status (0–1 vs. ≥2), and number of SG cycles (≤3 vs. >3), with comparisons using Fisher’s exact test given small cell counts and limited power.

The study was approved by the research ethics committee of each participating centre. Given its retrospective and observational nature, compliance with national regulations on data protection and clinical confidentiality was ensured in accordance with the General Data Protection Regulation (GDPR EU 2016/679) and Organic Law 3/2018 on the Protection of Personal Data and Guarantee of Digital Rights.

## 3. Results

### 3.1. Clinical Characteristics of the Study Population

**Figure 1:**
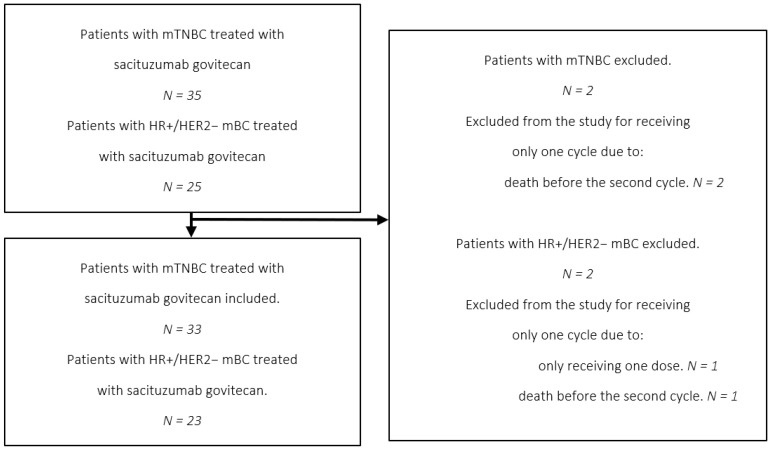
Flowchart of the selection process.

Of the 60 patients identified, 56 met the inclusion criteria. Among them, 33 patients (58.9%) had mTNBC, and 23 (41.1%) had HR+/HER2− mBC. The demographic and baseline characteristics are summarised in Table 1. Figure 1 illustrates the screening process and the patient selection flow.

#### 3.1.1. Demographic and Functional Data at the Start of Treatment

In the mTNBC group, the median age was 51.2 years (interquartile range [IQR] 45.9–60.5), whereas in the HR+/HER2− mBC group the median age was 62.2 years (IQR 55.7–65.4). Regarding ECOG performance status, most patients had an ECOG performance status of 1 in both the mTNBC (63.6%) and HR+/HER2− mBC (73.9%) groups. In the mTNBC group, 6 patients (18.2%) had an ECOG score ≥ 2, while in the HR+/HER2− mBC group, 3 patients (13.0%) had an ECOG of 2. At the end of the study period, 16 patients (48.5%) in the mTNBC group and 9 (39.1%) in the HR+/HER2− mBC group died. The detailed characteristics are presented in Table 1.

No dose reduction or treatment delays were observed. None of the patients required transfusions as post-SG treatment, and all patients received antiemetic therapy with netupitant 300 mg orally on days 1 and 8 of each cycle, according to the protocols of the participating hospitals (Supplementary Table S1). The median number of SG cycles administered was five (IQR: 3–8) in the mTNBC group and five (IQR: 3–9) in the HR+/HER2− mBC group. The median duration of SG treatment was 3.5 months (IQR: 2.02–5.48) for patients with mTNBC and 3.47 months (IQR: 1.87–6.53) for those with HR+/HER2− mBC (Supplementary Table S1 and Supplementary Figure S1).

#### 3.1.2. Location of Metastases

The most frequent metastatic sites in the mTNBC group were lymph nodes in 22 patients (66.7%), followed by bone in 15 patients (45.5%), and others, including pulmonary, hepatic, peritoneal, adrenal, mediastinal, muscular, and cutaneous involvement. Central nervous system (CNS) metastases were identified in 7 patients (21.2%) in the mTNBC group. In the HR+/HER2− mBC group, the most common metastatic sites were the bones (78.3%), liver (65.2%), and lungs (43.5%). Other sites included lymph nodes, pleura, peritoneum, skin, CNS, and gastroduodenal involvement (Table 1).

#### 3.1.3. Prior Treatments

In the mTNBC group, all patients (100%) had previously received taxanes, and most had been treated with platinum agents (93.9%), cyclophosphamide (69.7%), doxorubicin (69.7%), and capecitabine (54.5%). Other prior therapies included gemcitabine (36.4%), pembrolizumab (15.2%), bevacizumab (18.2%) and eribulin (12.1%).

In the HR+/HER2− mBC group, most patients received taxanes (95.7%), capecitabine (91.3%), cyclophosphamide (78.3%), and doxorubicin (73.9%). Previous endocrine therapies have been recorded, such as fulvestrant and letrozole, along with CDK4/6 inhibitors, particularly palbociclib. Prior anti-HER2 treatment was observed in both groups before SG initiation, although more frequently in the HR+/HER2− mBC group, with trastuzumab deruxtecan used in 13.0% of patients in this subgroup. The median number of prior treatments in the metastatic setting was three for mTNBC and four for HR+/HER2− mBC patients (Table 1).

### 3.2. Adverse Events and Use of G-CSF

#### 3.2.1. Observed Toxicity and Adverse Events

Multiple adverse events (AEs) were identified during SG treatment. The most common AEs in the mTNBC group were fatigue (48.5%), nausea and constipation (33.3%) and neutropenia (27.3%). In the HR+/HER2− mBC group, the most prevalent AEs were fatigue (56.5%), diarrhoea (26.1%), and alopecia (21.7%). Two cases (8.7%) of pneumonitis were reported in the HR+/HER2-mBC group. Table 2 provides a detailed overview of the AEs by group.

#### 3.2.2. Use of G-CSF and Neutropenia

In the mTNBC group, 28 patients (84.8%) received G-CSF prophylaxis, of whom nine developed neutropenia. None of the 6 patients who did not receive G-CSF experienced this event (Table 2). In the HR+/HER2− mBC group, G-CSF was administered to 17 patients (73.9%), and one case of neutropenia was identified in this subgroup. Among the six patients who did not receive G-CSF, one developed neutropenia (Table 2). Fisher’s exact test showed no statistically significant difference between G-CSF use and the incidence of neutropenia (*p* = 0.434). Descriptive subgroup analyses of neutropenia stratified by age, ECOG status, and number of SG cycles are provided in Supplementary Table S2 (HR+/HER2− mBC) and Table S3 (mTNBC). These analyses were exploratory and should be interpreted with caution.

### 3.3. Progression-Free Survival and Overall Survival

#### 3.3.1. Progression-Free Survival

**Figure 2:**
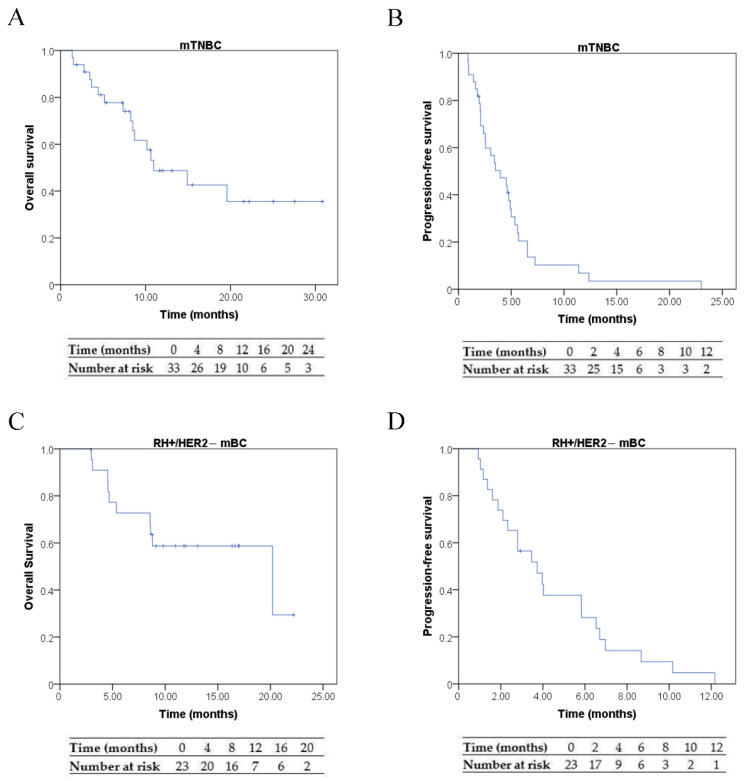
Survival outcomes in the mTNBC and RH+/HER2− mBC patient populations. Kaplan–Meier curves showing overall survival (OS) and progression-free survival (PFS), each with the corresponding number-at-risk table: (**A**) OS in mTNBC; (**B**) PFS in mTNBC; (**C**) OS in HR+/HER2− mBC; and (**D**) PFS in HR+/HER2− mBC.

The median progression-free survival (mPFS) in the mTNBC group was 4.0 months (95% CI, 1.94 to 5.98), with a standard error of 1.03. In the HR+/HER2− mBC group, mPFS was 3.7 months (95% CI, 2.02 to 5.44), with a standard error of 0.873. At the end of the study, disease progression was observed in 32 patients (93.9%) in the mTNBC group and in 22 patients (93.7%) in the HR+/HER2− mBC group. The corresponding Kaplan–Meier curves are shown in Figure 2.

#### 3.3.2. Overall Survival

In the mTNBC group, the median overall survival (mOS) was 11.0 months (95% CI, 4.8 to 17.1), with a standard error of 3.15. In the HR+/HER2− mBC group, mOS was 20.2 months (95% CI, 3.9 to 36.5), with a standard error of 8.3. At the end of the study, 48.5% of the patients with mTNBC and 43.5% of those with HR+/HER2− mBC died (Figure 2).

In the univariate analysis, variables potentially influencing OS (*p* < 0.1) included ECOG performance status, thrombocytopenia, and nausea as adverse events, and the presence of hepatic, gastroduodenal, pleural, and CNS metastases, as well as prior treatment with trastuzumab/pertuzumab. The univariate analysis curves for OS and PFS with and without CNS metastases are available for reference in Supplementary Figure S2.

In the multivariate analysis, no statistically significant differences were observed for ECOG performance status (*p* = 0.164), thrombocytopenia (*p* = 0.573), nausea (*p* = 0.292), CNS metastases (*p* = 0.061), or prior treatment with trastuzumab/pertuzumab (*p* = 0.258). In contrast, the presence of hepatic metastases had a significant impact on OS (HR: 7.76; 95% CI: 2.16–27.83; *p* = 0.002), as did gastroduodenal metastases (HR: 95.15; 95% CI: 7.20–1257.93; *p* = 0.002) and pleural metastases (HR: 9.59; 95% CI: 1.83–50.30; *p* = 0.008), all of which were associated with a significant reduction in OS (Table 3).

## 4. Discussion

SG, an ADC targeting Trop-2, has demonstrated efficacy and safety in the treatment of metastatic breast cancer (mBC), both in the mTNBC and HR+/HER2− subtypes. Our study provides additional evidence regarding the effectiveness and safety of SG in a real-world clinical setting, including patients with characteristics not represented in the pivotal ASCENT and TROPiCS-02 trials.

A notable difference observed in our cohort was related to the number of prior treatment lines received in both groups, which was higher than that in the ASCENT and TROPiCS-02 trials. In our study, patients received SG at more advanced lines of therapy, likely because of delays in the approval process for SG use in these mBC subtypes.

Regarding effectiveness, in the mTNBC group, the median PFS was 4.0 months and the median OS was 11.0 months—slightly lower than that reported in the phase III ASCENT trial (mPFS: 4.8 months; mOS: 11.8 months) [8]. This difference may partly reflect the more unfavourable characteristics of our cohort, in which 21.2% of patients had CNS metastases and 18.2% had an ECOG performance status ≥ 2, both of which were exclusion criteria in the ASCENT trial [8].

Several real-world studies have documented the effectiveness and safety of SG in patients with mTNBC, showing outcomes consistent with ours. A multicentre study conducted in the United Kingdom reported a higher mPFS (5.2 months vs. 4.0 months) but a lower mOS (8.7 months vs. 11.0 months) in a slightly older population (median age, 59 years) with lower ECOG scores and a lower rate of CNS metastases compared to our cohort (18% vs. 21.2%), which may explain these differences [13].

In a real-world study by Alaklabi et al., a similar mPFS was reported (4.8 months vs. 4.0 months in our population), with a slightly lower mOS (9.6 months vs. 11.0 months). It is worth noting that their population was older, with a median age of 60 years and a comparable prevalence of CNS metastases (21.7% vs. 21.2%) [14].

Another real-world multicentre study in patients with mTNBC, whose population had a median age of 53 years compared to 51.2 years in our cohort, and a higher proportion of CNS metastases (28.1% vs. 21.2%), showed similar survival outcomes, with an mPFS of 4.9 vs. 4.0 months and an mOS of 12.43 vs. 11.0 months [17].

Kalinsky et al., in a multicentre US-based cohort of mTNBC patients with a median age of 60 years and 17% with ECOG ≥ 2, reported survival outcomes with an mPFS of 3.8 months compared to 4.0 months in our study, and an mOS of 10 months vs. 11.0 months in our population [18].

A Spanish multicentre study on mTNBC patients assessing the real-world effectiveness and safety of SG reported outcomes very similar to ours, with a median PFS of 4.1 vs. 4.0 months and a median OS of 11.0 months in both cohorts [19].

In another real-world study conducted in Germany, slightly higher outcomes were observed, with a median PFS of 5.0 vs. 4.0 Â months and a median OS of 13.1 vs. 11.0 months, despite involving an older population (median age 57 vs. 51.2 years) and a comparable rate of CNS metastases (23.2% vs. 21.2%) [20].

Regarding the HR+/HER2− mBC subtype, the median OS in our cohort was 20.2 months, which is comparable to the 21 months reported in the Asian EVER-132-002 study and higher than the 14.4 months reported in the TROPiCS-02 trial. This difference may be partly explained by the low prevalence of brain metastases in our cohort (4.3%), as both trials excluded patients with this metastatic site. A previous study demonstrated that, in patients with HR+/HER2− mBC and brain metastases treated with SG, mPFS and mOS were reduced by 2.0 and 3.0 months, respectively [9,15,21].

However, the mPFS in our cohort was 3.7 months—lower than the 5.5 months reported for TROPiCS-02. This discrepancy may be due to the higher proportion of patients with ECOG ≥ 2 (13%), multiple prior lines of therapy, extensive visceral disease, and CNS metastases in our cohort [9]. In contrast, the mPFS of 3.7 months in our study was consistent with the 4.2 months observed in the EVER-132-002 trial, suggesting that real-world treatment of HR+/HER2− mBC may yield better OS outcomes than those reported in the phase III TROPiCS-02 trial.

In a multicentre real-world study of a cohort that included both mTNBC and HR+/HER2− mBC patients treated with SG, those with HR+/HER2− mBC showed an mPFS of 6.1 months and an mOS of 12.5 months, reflecting a higher mPFS but lower mOS compared to our study, yet aligned with the outcomes of TROPiCS-02 [16].

While our study confirms the effectiveness of SG in a real-world setting, the slight differences in mPFS and mOS compared with the ASCENT and TROPiCS-02 trials underscore the variability of clinical outcomes outside controlled environments, particularly in heavily pretreated patients with CNS metastases and ECOG ≥ 2. Nonetheless, our mPFS results, although slightly lower, are consistent with other real-world studies, and our OS findings are comparable to or even higher than those from the TROPiCS-02 trial, especially in the case of HR+/HER2− mBC, where they align well with both real-world data and the EVER-132-002 study.

Our multivariate analysis results indicate that the presence of hepatic, pleural, and gastroduodenal metastases is a poor prognostic factor for overall survival (OS). The unfavourable prognosis associated with hepatic metastases is consistent with findings from a real-world study of patients with mTNBC and HR+/HER2− mBC treated with SG [16]. Notably, the same study did not find a statistically significant association between CNS metastases and reduced OS, mirroring our own findings, which may suggest that treatment with SG is not necessarily affected by this type of metastatic involvement. However, further studies are required to establish this association. Lastly, the findings regarding pleural and gastroduodenal metastases in our multivariate analysis should be interpreted with caution, given the low number of patients with these metastatic sites (two and one patient, respectively).

In terms of safety, SG treatment for both mTNBC and HR+/HER2− mBC showed a toxicity profile consistent with clinical trials, real-world studies, and safety analyses of SG, although with a lower incidence of adverse events, such as alopecia, anaemia, neutropenia, diarrhoea, and nausea [8,9,14,22]. Fatigue, neutropenia, nausea, and constipation were the most common adverse events. Adverse effects not previously reported in other studies, such as xerostomia and xerophthalmia, were identified in both groups. No severe adverse events (AEs) such as neutropenic colitis or septic shock were observed; these are high-priority AEs owing to their impact on patients, with reported fatality rates of 31.3% and 77.8%, respectively [22].

As certain toxicities, particularly neutropenia and diarrhoea, have been associated with the reduced metabolism of SN-38 in patients carrying specific UGT1A1 polymorphisms, we considered the potential influence of this pharmacogenetic factor in our cohort. Genotype data for UGT1A1 were not available for our patients, as according to the United States Prescribing Information issued by the Food and Drug Administration (FDA) (FDA Trodelvy label link, https://www.accessdata.fda.gov/drugsatfda_docs/label/2021/761115s009lbl.pdf) (accessed on 11 August 2025) and the Summary of Product Characteristics from the European Medicines Agency (EMA) (EMA Trodelvy Summary of Product Characteristics link, https://www.ema.europa.eu/en/documents/product-information/trodelvy-epar-product-information_en.pdf) (accessed on 11 August 2025), routine determination of the UGT1A1 *28 polymorphism is not recommended, and no specific dose adjustments are indicated in its presence. Conversely, the Summary of Product Characteristics for SG issued by the Spanish Agency for Medicines and Health Products (AEMPS) acknowledges an increased risk of adverse reactions in UGT1A1 *28/*28 homozygous patients and advises close monitoring, although testing is not required prior to treatment initiation (Technical Sheet Trodelvy AEMPS, https://cima.aemps.es/cima/dochtml/ft/1211592001/FT_1211592001.html) (accessed on 11 August 2025).

Despite this, determination of the genotype of UGT1A1 could have been relevant to contextualise the incidence of diarrhoea and neutropenia, since evidence from the TROPiCS-02 trial has shown that patients homozygous for UGT1A1 *28/*28 had higher rates of grade 3 or 4 diarrhoea and neutropenia than other subgroups [9].

Moreover, a recent study observed that the presence of the UGT1A1 *28/*28 polymorphism defines a pharmacogenetic profile characterised by reduced metabolism of SN-38, resulting in a significant increase in treatment discontinuations due to toxicity (HR 5.52; 95% CI 1.15–26.49; *p* = 0.03), mainly because of febrile neutropenia (FN) and diarrhoea, although without a statistically significant impact on discontinuation due to disease progression (HR 0.80; 95% CI 0.39–1.65; *p* = 0.54). These data suggest the clinical utility of UGT1A1 genotyping in optimising dose management and preventing adverse events in patients treated with SG [23].

The use of G-CSF as primary prophylaxis was high (84.8% in mTNBC; 73.9% in HR+/HER2− mBC), with a lower prevalence of neutropenia of any grade (27.3% in mTNBC and 8.7% in HR+/HER2− mBC) than that reported in other mTNBC studies, where the prevalence ranged from 55% to 66.7%, depending on the study and the rate of G-CSF use [8,12,13,15]. In two real-world studies, one in the United States and one in Germany, in patients with mTNBC, the incidence of neutropenia of any grade was 33.0% and 32.6%, respectively, similar to our findings. In the first study, G-CSF was administered to 58.0% of patients, while in the second study, primary G-CSF prophylaxis on days 2, 3, and 4 of each cycle was administered to 25.6% of patients [17,19].

In the ASCENT and TROPiCS-02 trials, the incidence of neutropenia was 66.7% and 71%, respectively, whereas FN occurred in only 6.1% and 5% of patients, respectively. This places SG in the low-to-intermediate FN risk category according to the EORTC, SEOM, and ESMO criteria, which recommend primary G-CSF prophylaxis when the FN risk exceeds 20% or between 10% and 20% in the presence of risk factors such as advanced age, comorbidities, or curative treatment intent [8,9,24,25,26].

Nevertheless, the ongoing phase II PRIMED study is exploring prophylactic G-CSF administration at a dose of 0.5 MU/kg/day on days 3, 4, 10, and 11 in patients receiving SG. Preliminary results have shown a reduction in the incidence of grade ≥ 3 neutropenia from 63% to 28% during the first two cycles [27]. Despite the differences in G-CSF dosing between the PRIMED study and our own, the incidence of neutropenia was similar (27.3% in our study vs. 28% in PRIMED). The results obtained from Fisher’s exact test showed no statistically significant differences between patients who did and did not receive G-CSF. Interestingly, contradictory findings were observed: in the mTNBC group, a higher percentage of neutropenia cases occurred among those who received prophylaxis than among those who did not, whereas in the HR+/HER2− mBC group, neutropenia was more frequent in patients who had not received primary prophylaxis. Further studies with larger patient cohorts are needed to establish the role of prophylactic G-CSF in preventing SG-induced neutropenia.

This difference in the incidence of any-grade neutropenia among SG-treated patients receiving primary prophylaxis has also been observed in another study, in which 20% of patients received primary prophylaxis and 59% received G-CSF. In that study, the incidence of any-grade neutropenia was 25% in patients who received primary and/or secondary prophylaxis compared to 44% in those who did not receive prophylaxis [28]. It is important to highlight that serious AEs related to neutropenia, such as neutropenic colitis or sepsis, and other myelosuppression-related complications, such as septic shock, have been reported with SG [29].

Further studies with larger patient cohorts are needed to determine whether primary G-CSF prophylaxis effectively reduces the total incidence of neutropenia and the associated AEs in patients treated with SG.

As for the limitations of our study, its retrospective design introduces potential selection and information biases, as it relies on a clinical record review without a prospective protocol to ensure standardised variable monitoring, limiting the ability to draw firm causal inferences. The small sample size reduced statistical power, hampering the detection of clinically meaningful differences.

The objective response rate (ORR) was not determined, as it is dependent on the request for imaging studies which, due to the high patient workload, are not routinely performed in the everyday clinical practice of the participating hospitals. The absence of ORR data may lead to limitations such as measurement and information bias or loss of comparability with controlled clinical trials in which ORR is assessed.

The lack of systematic assessment of objective response according to the RECIST 1.1 criteria introduces heterogeneity in tumour response evaluation and inter-observer variability, which may lead to measurement bias. Similarly, the absence of data on dose reductions and adjustments limits the ability to comprehensively analyse the dose–response–toxicity relationship.

We also report exploratory subgroup findings for neutropenia (Supplementary Tables S2 and S3); however, these analyses were underpowered and primarily descriptive.

## 5. Conclusions

This retrospective multicentre study provides evidence on the effectiveness and safety of SG in the treatment of metastatic breast cancer (mBC), both in patients with triple-negative breast cancer and in those with HR+/HER2− disease. Our findings, although slightly lower in terms of mPFS and mOS for the mTNBC subtype compared to pivotal clinical trials, are consistent with results from other real-world studies. Moreover, in the HR+/HER2− mBC group, a higher mOS was observed than in the pivotal TROPiCS-02 trial, confirming that SG retains clinically meaningful effectiveness even in patients with advanced ECOG status, extensive visceral disease, or CNS metastases in the HR+/HER2− mBC setting.

Toxicity was consistent with that reported in previous studies and in phase III clinical trials that led to the approval of SG for mTNBC and HR+/HER2− mBC, with fatigue, neutropenia, nausea, and constipation being the most frequent adverse events. In our cohort, the use of G-CSF as primary prophylaxis did not show a statistically significant association with a reduced incidence of neutropenia.

## Figures and Tables

**Table 1:** Population characteristics, metastatic sites, and previous treatment.

Variable	mTNBC (*n* = 33)	RH+/HER2− mBC (*n* = 23)
Patients, No. (%)	33 (58.9%)	23 (41.1%)
Exitus, No. (%)	16 (48.5%)	9 (39.1%)
Age (RIC)	51.2 (45.9–60.5)	62.2 (55.7–65.4)
Gender, No. (%)		
Female	33 (100%)	23 (100%)
ECOG, No. (%)		
ECOG 0	6 (18.2%)	3 (13.0%)
ECOG 1	21 (63.6%)	17 (73.9%)
ECOG 2	5 (15.2%)	3 (13%)
ECOG 3	1 (3%)	0 (0%)
Metastatic location, No. (%)		
Bone	15 (45.5%)	18 (78.3%)
Liver	12 (36.4%)	15 (65.2%)
Pulmonary	10 (30.3%)	10 (43.5%)
Lymph node	22 (66.7%)	9 (39.1%)
CNS	7 (21.2%)	1 (4.3%)
Peritoneal	1 (3%)	2 (8.6%)
Adrenal	1 (3%)	0 (0%)
Mediastinal	2 (6.1%)	0 (0%)
Muscle	2 (6.1%)	0 (0%)
Cutaneous	2 (6.1%)	2 (8.6%)
Pleural	0 (0%)	2 (8.6%)
Gastroduodenal	0 (0%)	1 (4.3%)
Previous lines of therapy in the metastatic setting, No. (%)		
≤3	25 (75.8%)	4 (17.4%)
>3	8 (24.2%)	19 (82.6%)
Previous chemotherapy drug, No. (%)		
Taxane	33 (100%)	22 (95.7%)
Platinum	31 (93.9%)	6 (26.1%)
Cyclophosphamide	23 (69.7%)	18 (78.3%)
Doxorubicin	23 (69.7%)	17 (73.9%)
Capecitabine	18 (54.5%)	21 (91.3%)
Gemcitabine	12 (36.4%)	3 (13%)
Eribulin	4 (12.1%)	14 (60.9%)
Previous anti-VEGF treatment, No. (%)		
Bevacizumab	6 (18.2%)	4 (17.4%)
Previous immune checkpoint inhibitor treatment, No. (%)		
Pembrolizumab	5 (15.2%)	0 (0%)
Previous CDK4/6 inhibitor treatment, No. (%)		
Palbociclib	0 (0%)	11 (47.8%)
Ribociclib	0 (0%)	2 (8.7%)
Abemaciclib	0 (0%)	1 (4.3%)
Previous hormone therapy, No. (%)		
Letrozol	0 (0%)	9 (39.1%)
Tamoxifen	0 (0%)	4 (17.4%)
Fulvestrant	0 (0%)	9 (39.1%)
Previous anti-HER2 therapies, No. (%)		
Trastuzumab/Pertuzumab	2 (6.1%)	1 (4.3%)
Trastuzumab deruxtecan	1 (3.0%)	3 (%13.0%)
Previous everolimus/exemestane therapy, No. (%)	0 (0%)	6 (26.1%)

**Table 2:** Adverse effects and use of G-CSF.

Adverse Events/Variable	mTNBC (*n* = 33)	RH+/HER2− mBC (*n* = 23)
AEs (any degree), No. (%)		
Fatigue	16 (48.5%)	13 (56.5%)
Constipation	11 (33.3%)	5 (21.7%)
Nausea	11 (33.3%)	5 (21.7%)
Neutropenia	9 (27.3%)	2 (8.7%)
Diarrhoea	7 (21.2%)	6 (26.1%)
Alopecia	7 (21.2%)	5 (21.7%)
Dysgeusia	6 (18.2%)	1 (4.3%)
Xerostomia	6 (18.2%)	4 (17.4%)
Mucositis	5 (15.2%)	3 (13.0%)
Hyporexia	5 (12.2%)	3 (13.0%
Abdominal pain	2 (6.1%)	0 (0%)
Anaemia	1 (3.0%)	1 (4.3%)
Thrombocytopenia	1 (3.0%)	1 (4.3%)
Hypersensitivity	1 (3.0%)	0 (0%)
Headache	1 (3.0%)	0 (0%)
Pneumonitis	0 (0%)	2 (8.7%)
Xerophthalmia	0 (0%)	1 (4.3%)
Onychomycosis	0 (0%)	1 (4.3%)
Malleolar oedema	0 (0%)	1 (4.3%)
Prophylactic administration of G-CSF, No. (%)	28 (84.8%)	17 (73.9%)
Neutropenia with G-CSF	9 (32.1%)	1 (5.9%)
Neutropenia without G-CSF	0 (0%)	1 (16.7%)

**Table 3:** Multivariate analyses for overall survival.

Variable	Univariate Analysis	Multivariate Analysis	
	*p*-Value	HR (95% CI)	*p*-Value
ECOG			
0	<0.001	2.14 (0.73–6.24)	0.164
1			
2			
3			
AEs			
Thrombocytopenia	0.001	0.46 (0.03–6.71)	0.573
Nausea	0.05	1.76 (0.61–5.03)	0.292
Metastatic location			
Hepatic	0.035	7.76 (2.16–27.83)	0.002
CNS	0.004	4.02 (0.94–17.25)	0.061
Pleural	0.005	9.59 (1.83–50.30)	0.008
Gastroduodenal	0.052	95.15 (7.20–1257.93)	0.002
Previous chemotherapy drug			
Trastuzumab/Pertuzumab	0.017	2.65 (0.49–14.38)	0.258

## Data Availability

The data that support the findings of this study are not openly available owing to reasons of sensitivity and are available from the corresponding author upon reasonable request. Data are located in controlled-access data storage at the Hospital General Universitario Santa Lucía.

## Supplementary Materials

The following supporting information can be downloaded at https://www.mdpi.com/article/10.3390/biomedicines13092059/s1: Figure S1: Swimmer plot showing the time on sacituzumab govitecan (SG) treatment for each patient with metastatic triple-negative breast cancer (mTNBC, blue) or HR+/HER2− metastatic breast cancer (HR+/HER2− mBC, orange); Figure S2: Kaplan–Meier curves for (A) overall survival (OS) and (B) progression-free survival (PFS) according to the presence (green line) or absence (blue line) of central nervous system (CNS) metastases in the overall study population; Table S1: Summary of sacituzumab govitecan (SG) treatment characteristics in patients with metastatic triple-negative breast cancer (mTNBC) and HR+/HER2− metastatic breast cancer (HR+/HER2− mBC); Table S2: Descriptive subgroup analysis of neutropenia incidence in patients with HR+/HER2− metastatic breast cancer, stratified by age (<60 vs. ≥60 years), ECOG performance status (0–1 vs. ≥2), and number of SG cycles received (≤3 vs. >3); Table S3: Descriptive subgroup analysis of neutropenia incidence in patients with metastatic triple-negative breast cancer (mTNBC), stratified by age (<60 vs. ≥60 years), ECOG performance status (0–1 vs. ≥2), and number of SG cycles received (≤3 vs. >3).

## Abbreviations

The following abbreviations are used in this manuscript:

AEMPS	Spanish Agency for Medicines and Health Products
AEs	Adverse Events
CDK	Cyclin-Dependent Kinase
CNS	Central Nervous System
ECOG	Eastern Cooperative Oncology Group
EMA	European Medicines Agency
ER	Oestrogen Receptor
ET	Endocrine Treatment
FDA	Food and Drug Administration
FN	Febrile Neutropenia
G-CSF	Granulocyte Colony-Stimulating Factor
HER2	Human Epidermal Growth Factor Receptor 2
HR	Hazard Ratio
IA	Aromatase Inhibitors
IHC	Immunohistochemistry
IQR	Interquartile Range
mBC	Metastatic Breast Cancer
mOS	Median Overall Survival
mPFS	Median Progression-free Survival
mTNBC	Metastatic Triple-Negative Breast Cancer
PR	Progesterone Receptor
SG	Sacituzumab Govitecan
Trop-2	Trophoblast Cell Surface Antigen 2
