# Supplementary material for: Effectiveness and Safety of Sacituzumab Govitecan in Real-World Clinical Practice in Patients with Metastatic Triple-Negative and HR+/HER2-Negative Breast Cancer
Source: Biomedicines. 2025 Aug 23;13(9):2059. doi: 10.3390/biomedicines13092059 (PMC12467514; doi:10.3390/biomedicines13092059)
Supplement: Supplementary file 1 [file biomedicines-13-02059-s001.zip › biomedicines-3805541-supplementary.pdf]

biomedicines

Supplementary material

Effectiveness and Safety of Sacituzumab Govitecan in Real-World Clinical Practice in Patients with Metastatic Triple-Negative and HR+/HER2-Negative Breast Cancer

Fernando Lago-Ballester [1,2], Adrián Martínez-Orea [3], Ana Laorden-Carrasco [4], María Sacramento Díaz-Carrasco [4], José Carlos Titos-Arcos [3], María Carmen Mira-Sirvent [1], Ginés Luengo-Gil [2,5,*] and Mónica Martínez-Penella [1,2,5,*]

1 Hospital Pharmacy Department, Hospital General Universitario Santa Lucía, 30202 Cartagena, Spain; fernando.lago@carm.es (F.L.-B.); mariac.mira@carm.es (M.C.M.-S.)
2 Health Sciences Faculty, Universidad Católica de Murcia (UCAM), 30107 Guadalupe, Spain
3 Hospital Pharmacy Department, Hospital General Universitario Morales Meseguer, 30008 Murcia, Spain; adrian.martinez6@carm.es (A.M.-O.); josec.titos@carm.es (J.C.T.-A.)
4 Hospital Pharmacy Department, Instituto Murciano de Investigación Biosanitaria Pascual Parrilla (IMIB), Hospital Clínico Universitario Virgen de La Arrixaca, 30120 Murcia, Spain; ana.laorden@carm.es (A.L.-C.); msacramento.diaz@carm.es (M.S.D.-C.)
5 Group of Molecular Pathology and Pharmacogenetics, Hospital Pharmacy and Pathology Department, Instituto Murciano de Investigación Biosanitaria (IMIB), Hospital General Universitario Santa Lucía, 30202 Cartagena, Spain
* Correspondence: gluengo@ucam.edu (G.L.-G.); monica.martinez4@carm.es (M.M.-P.); Tel.: +34-968128602 (ext. 951439) (G.L.-G.)

biomedicines

MDPI

1. Supplementary material

Subtype	Median cycles administered (IQR)	Median treatment duration, months (IQR)	Dose reductions recorded	Treatment delays recorded	Post-SG transfusions required	Antiemetic therapy
mTNBC	5 (3–8)	3.5 (2.02–5.48)	No	No	No	Netupitant 300 mg orally days 1 and 8 of each cycle (per hospital protocol)
HR+/HER2− mBC	5 (3–9)	3.47 (1.87–6.53)	No	No	No	Netupitant 300 mg orally days 1 and 8 of each cycle (per hospital protocol)

Supplementary Table S1. Summary of sacituzumab govitecan (SG) treatment characteristics in patients with metastatic triple-negative breast cancer (mTNBC) and HR+/HER2− metastatic breast cancer (HR+/HER2− mBC). Data are expressed as median (interquartile range [IQR]) unless otherwise indicated.

HR+/HER2− mBC

Neutropenia	AGE		*p*	ECOG		*p*	CYCLES		*p*
	<60	≥60		0–1	≥2		≤3	>3	
No	8	13	1.000	18	3	1.000	8	13	0.526
Yes	1	1		2	0		0	2	

Supplementary Table S2. Descriptive subgroup analysis of neutropenia incidence in patients with HR+/HER2− metastatic breast cancer, stratified by age (<60 vs ≥60 years), ECOG performance status (0–1 vs ≥2), and number of SG cycles received (≤3 vs >3). *p*-values were calculated using Fisher’s exact test.

mTNBC

Neutropenia	AGE		*p*	ECOG		*p*	CYCLES		*p*
	<60	≥60		0–1	≥2		≤3	>3	
No	18	6	1.000	18	5	0.648	8	16	1.000
Yes	7	2		8	1		3	6	

Supplementary Table S3. Descriptive subgroup analysis of neutropenia incidence in patients with metastatic triple-negative breast cancer (mTNBC), stratified by age (<60 vs ≥60 years), ECOG performance status (0–1 vs ≥2), and number of SG cycles received (≤3 vs >3). p-values were calculated using Fisher’s exact test.

biomedicines

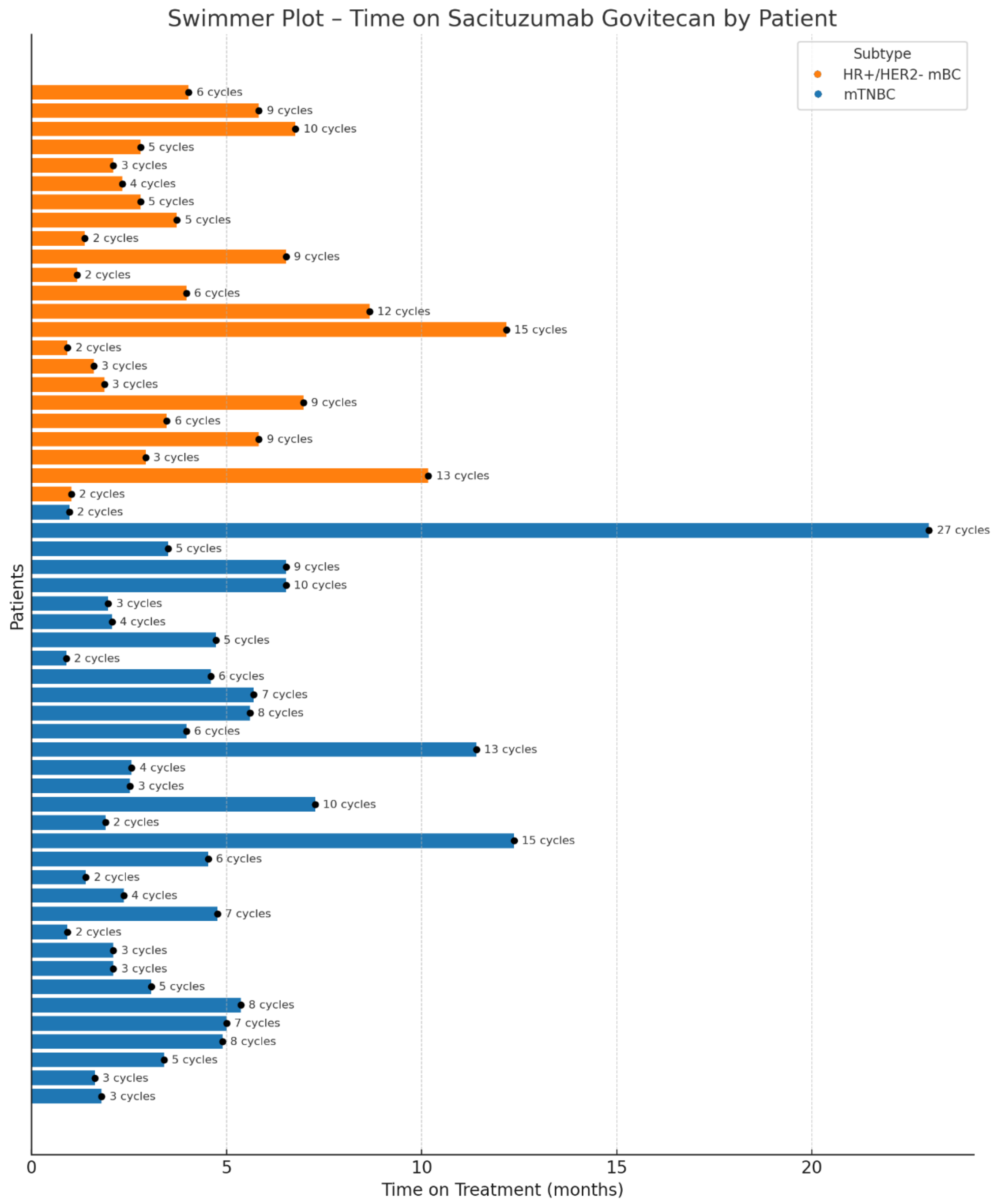

Supplementary Figure S1. Swimmer plot showing the time on sacituzumab govitecan (SG) treatment for each patient with metastatic triple-negative breast cancer (mTNBC, blue) or HR+/HER2− metastatic breast cancer (HR+/HER2− mBC, orange). Each bar represents the treatment duration for an individual patient, with a black circle indicating the end of the follow-up or disease progression. The numbers to the right of each bar indicate the total number of cycles administered.

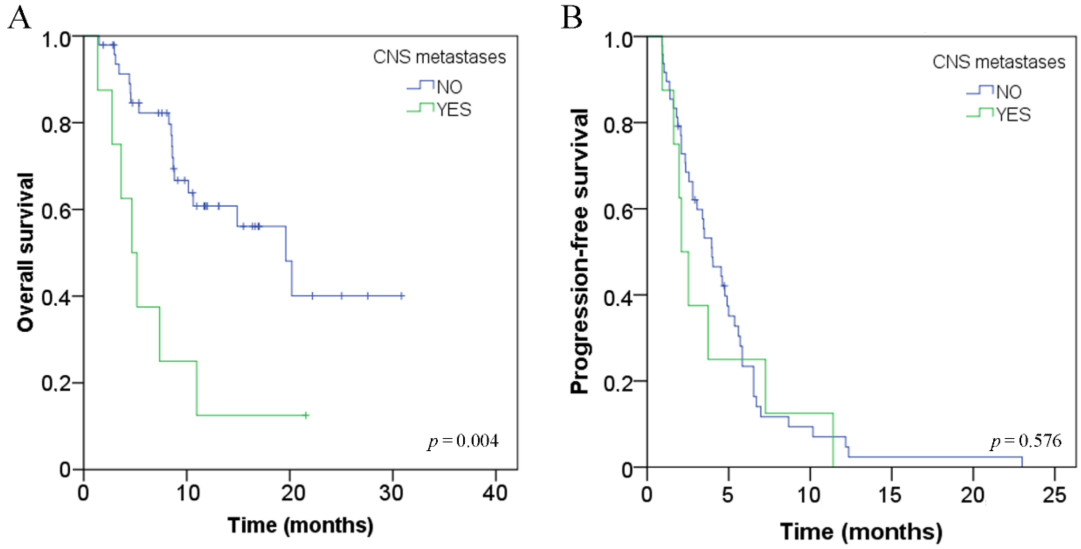

Supplementary Figure S2. Kaplan–Meier curves for (A) overall survival (OS) and (B) progression-free survival (PFS) according to the presence (green line) or absence (blue line) of central nervous system (CNS) metastases in the overall study population. Median OS was 4.6 months in patients with CNS metastases versus 19.6 months in those without CNS metastases (log-rank $p = 0.004$). Median PFS was 2.1 versus 3.9 months, respectively (log-rank $p = 0.576$).
